# Ultrasonographic examination of the forestomachs and the abomasum in ruminal drinker calves

**DOI:** 10.1186/1751-0147-55-1

**Published:** 2013-01-08

**Authors:** Ueli Braun, Alexandra Gautschi

**Affiliations:** 1 Department of Farm Animals, Vetsuisse Faculty, University of Zurich, Zurich, Switzerland

## Abstract

**Background:**

The study investigated the ultrasonographic appearance of the reticulum, rumen, omasum and abomasum of calves with ruminal drinking syndrome.

**Methods:**

In ten milk-fed calves with ruminal drinking syndrome the reticulum, rumen, omasum and abomasum were examined by ultrasonography using a 5-MHz linear transducer before, during and after the ingestion of milk.

**Results:**

The reticulum could be imaged in eight of ten calves before feeding. The reticular wall appeared as an echoic line, similar to mature cattle, and reticular folds were seen in eight calves. The reticular content appeared as echoic heterogeneous fluid. Reticular contractions were biphasic with 1.0 ± 0.38 contractions per minute. The rumen had a mean wall thickness of 2.1 mm dorsally, 3.5 mm at the level of the longitudinal groove, and 3.2 mm ventrally. The ventral sac of the rumen of all calves contained echoic heterogeneous liquid. During feeding the milk entering the rumen could be seen as hyperechoic liquid in five calves. The omasum was seen on the right side as a crescent-shaped line medial to the liver in seven calves. Only the omasal wall closest to the transducer was seen as an echoic line with a mean thickness of 2.7 mm. The ultrasonographic appearance of the omasum did not change during or after feeding. The abomasum was seen immediately caudal to the xyphoid on both sides of the midline before feeding. The mean length at the ventral midline was 22.2 cm. The ingesta were heterogeneous in all calves and the abomasal folds were distinct in eight. The mean lateral expansion of the abomasum from the ventral midline to the left and right varied from 8.7 to 13.8 cm and from 4.3 to 11.3 cm. The milk entering the abomasum was observed in all calves, and signs of milk clotting were seen in all calves 15 minutes after feeding.

**Conclusion:**

This study showed that ultrasonography is useful for detecting milk in the reticulum and rumen of calves with ruminal drinking syndrome.

## Background

Ruminal drinking syndrome is a type of chronic indigestion of calves caused by failure of the reticular groove, administration of milk via stomach tube or reflux of milk from the abomasum into the rumen [1]. In the rumen, milk undergoes bacterial fermentation, which involves the transformation of lactose into lactate by lactobacilli causing ruminal and metabolic acidosis [1,2]. Even in normal calves, closure of the reticular groove is not complete, and about 10% of the ingested milk reaches the rumen [3]. This has no adverse effects and the milk is moved to the abomasum within three hours [4]. Because of butyric and lactic acid accumulation in the epithelium of the reticulorumen, ruminal drinking leads to hyperkeratotic parakeratosis and severe reticuloruminitis accompanied by epithelial loss, erosions and necrosis [1]. A diagnosis of ruminal drinking is based on finding yellowish-white and sour-smelling ruminal fluid with a pH of 4.0 to 5.0 [1,5]. To the best of our knowledge, ultrasonographic findings in calves with this syndrome have not been published, although ultrasonography is an established imaging technique for examination of the intestinal tract in cattle. There have been several reports on ultrasonography of the reticulum [6-8], rumen [9], omasum [10] and abomasum [11] of healthy mature cattle. There have also been many studies on the ultrasonographic findings in various gastrointestinal disorders in cattle, which were summarized in review articles [12,13]. Ultrasonographic studies of the forestomachs and abomasum in calves include abdominal findings in newborn calves [14], the volume, location and emptying rate of the abomasum of milk-fed calves [15], the effect of erythromycin, neostigmine and metoclopramide on abomasal motility and emptying rate [16] and clotting of milk in the abomasum of calves [17,18]. The ultrasonographic findings in healthy calves before, during and after the ingestion of milk have recently been published [19,20]. The goal of the present study was to describe the ultrasonographic findings of the reticulum, rumen, omasum and abomasum in calves with ruminal drinking syndrome and to determine whether ultrasonography is useful for the diagnosis of this condition.

## Methods

The study protocol was approved by the Animal Care Committe of the Canton of Zurich, Switzerland.

### Animals

Ten milk-fed calves 13 to 90 days old (mean ± sd = 31.7 ± 24.12 days) and weighing 39 to 92 kg (60.2 ± 16.80 kg) with ruminal drinking syndrome were used. The rumen content of all calves resembled milk and had a sour smell (for details see Results). There were three male and seven female calves and breeds consisted of four Swiss Braunvieh, four cross-bred beef, one Simmental and one Jersey. All calves were tested negative for BVD antigen.

### Clinical examination

The general condition and mental status, rectal temperature and heart and respiratory rates were assessed and the lungs, rumen and intestines were auscultated. In all calves, blood was collected for haematological, serum biochemical and venous blood gas analysis. Voided urine was collected for determination of specific gravity and examination with a test strip (Combur Test®, Roche Pharma), and samples of ruminal fluid, collected using a stomach tube, were assessed for colour, smell, consistency and pH.

### Ultrasound machine and video recorder

A real-time scanner (EUB 8500, Hitachi Medical Systems) and a 5.0-MHz linear transducer (Type EUP L53) were used to scan the calves. The ultrasound machine was connected to a video recorder (Panasonic DVC Pro Digital, Osaka) to enable continuous recording of the images before, during and after the intake of milk.

### Preparation of calves

The hair was clipped on both sides and in the ventral region. Alcohol was used to remove oily secretions from the skin, and contact gel was applied to the skin.

### Feeding of milk

After a ten-hour fast, the calves were fed 2 litres of cow’s milk warmed to 39°C using a nipple connected to a rubber hose, which reached to bottom of a milk bucket.

### Ultrasonographic examinations

The reticulum, rumen, omasum and abomasum were evaluated before, during and 15 minutes after feeding. Because of the large number of examinations, they were carried out in four sessions over a period of two days as follows:

- Day 1, morning: examination of reticulum, rumen, omasum and abomasum before feeding. The reticulum was re-examined 15 minutes after feeding.

- Day 1, afternoon: examination of the rumen during feeding and 15 minutes later.

- Day 2, morning: examination of the omasum during feeding and 15 minutes later.

- Day 2, afternoon: examination of the abomasum during feeding and 15 minutes later.

### Ultrasonography of the reticulum

The shape, contour, wall, wall thickness and contents of the reticulum were assessed by scanning the left ventral thoracic region. Reticular motility was recorded on video during nine minutes and analysed later as described previously [6,8]. The number of reticular contractions was recorded, and the time between contractions was measured using a stopwatch. In each calf, the durations of the two contractions of one biphasic contraction were measured using a stopwatch. The duration of the first reticular contraction was defined as the time from the first contractile movement of the reticulum to the end of its incomplete relaxation. The second contraction, which immediately followed the first, was timed from the end of incomplete relaxation to when the reticulum returned to its original location. The amplitude of the first contraction and the displacement of the reticulum after partial relaxation following the first contraction were measured using an electronic ruler placed along the direction of the contraction. The time required for the first contraction was determined and the contraction speed calculated.

### Ultrasonograpy of the rumen

The rumen was assessed by scanning the 7th to 12th intercostal spaces and the flanks on both sides from dorsal to ventral with the transducer held parallel to the ribs. The location of the rumen in the abdomen and visualisation of the cranial dorsal blind sac, ventral and dorsal sacs of the rumen and ruminal wall and contents were evaluated. The content of the rumen was assessed in the dorsal sac of the rumen, at the level of the longitudinal groove and in the ventral sac of the rumen. The dorsal and ventral visible limits of the rumen were determined by measuring their distance from the midline of the back, analogous to the technique described in goats [21]. The size of the rumen was calculated by subtracting the measurement of the dorsal limit of the rumen from that of the ventral limit. The location of the longitudinal groove was also measured by determining its distance from the midline of the back. The dorsal sac of the rumen extended from the dorsal limit of the rumen to the longitudinal groove, and the ventral sac extended from the longitudinal groove to the ventral limit of the rumen. The thickness of the wall was measured in the region of the dorsal sac, longitudinal groove and ventral sac of the rumen using the electronic cursors. During and after feeding, the rumen was assessed only from the left flank.

### Ultrasonography of the omasum

The omasum was evaluated by scanning the intercostal spaces on the right from cranial to caudal and from dorsal to ventral with the transducer held parallel to the ribs. Visualisation of the wall, leaves and content of the omasum was assessed. The omasum was observed for four minutes to determine whether omasal contractions occurred. The thickness of the omasal wall was measured using the electronic cursors. The location and size of the omasum were then assessed in the same manner as the rumen by determining the dorsal and ventral limits.

### Ultrasonography of the abomasum

The abomasum was assessed from the 7th to 12th intercostal spaces and the flanks on both sides by holding the transducer parallel to the ribs and moving it dorsally from the ventral midline. The location and size of the abomasum as well as visualisation of the wall, folds and content of the abomasum were evaluated. The thickness of the abomasal wall was measured using the electronic cursors. From the ventral midline, the distance from the xyphoid to the cranial margin of the abomasum was measured as well as the length of the abomasum. During feeding, a 4-minute video recording was made for later analysis. The occurrence of milk clotting was assessed using published criteria [15,17]. The size of the solid content was grouped as 0.5 to 1.0 cm, 1.01 to 5.0 cm, or larger than 5 .0 cm. The relationship between solid and liquid content was grouped as mostly liquid, equal proportions of solid and liquid, or mostly solid content.

### Statistical analysis

Frequencies, means and standard deviations were calculated using the statistical software program STATA 10 (StataCorp LP, Collage Station, Texas, USA). Differences were analysed using an analysis of variance (ANOVA) for repeated measures, and means were compared using a paired *t*-test. A value of P < 0.05 was considered significant.

## Results

### Clinical findings

The general condition and mental status were impaired in all calves. The rectal temperature ranged from 36.8 to 39.7°C (mean ± sd = 38.6 ± 0.88°C), the heart rate from 60 to 120 bpm (93.6 ± 17.81 bpm) and the respiratory rate from 20 to 44 breaths per minute (29.6 ± 7.82 breaths per minute). All calves had reduced skin turgor, eight had a cold and dry muzzle, six had injected scleral vessels and five had increased respiratory sounds due to bronchopneumonia. There was no ruminal motility. Swinging auscultation and simultaneous auscultation and percussion yielded splashing sounds and high-pitched metallic sounds (steel band), respectively, on the left in all calves and on the right in three calves. The faeces were watery in four calves and of semi-solid consistency in four others.

### Complete blood cell count, blood biochemistry and examination of urine and ruminal fluid

These findings were described in detail elsewhere [19]. The most important abnormal findings were leukocytosis (mean, min., max. = 17.5, 10.5, 47.0 × 10^9^ leukocytes/l blood), increased fibrinogen concentration (7.8, 2.0, 19.0 g/l), azotaemia (urea, 14.1, 2.9, 50.7 mmol/l) and metabolic acidosis with a blood pH of 7.2 (min. 6.8, max. 7.4) and base excess of −10.3 (min. -25.0, max. 9.8 mmol/l). The urine was normal in all calves. The ruminal fluid had a milky appearance in all calves, an acidic odour and a pH ranging from 3.5 to 8.0 (mean 5.5).

### Ultrasonography of the reticulum

**Figure 1:**
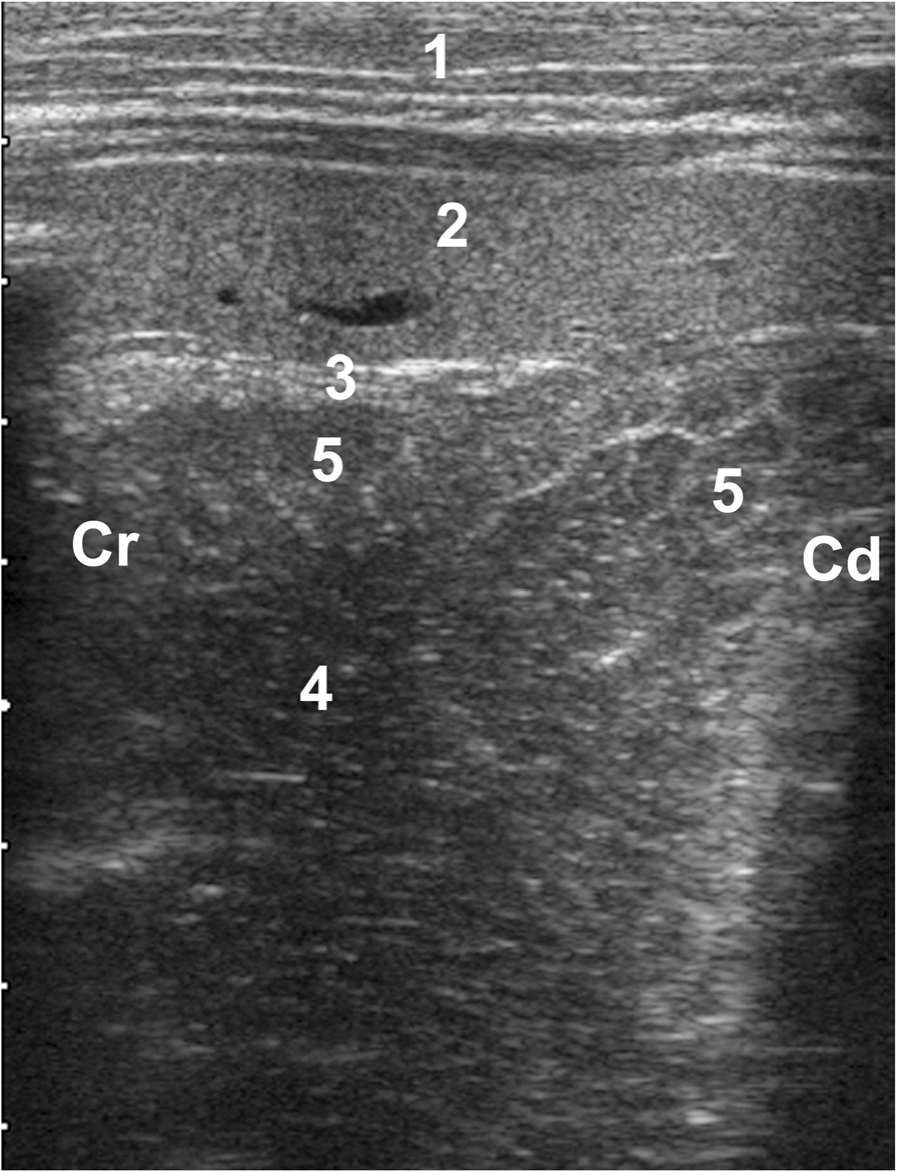
**Ultrasonogram of the reticulum.** Ultrasonogram of the reticulum of a 37-day-old Swiss Braunvieh calf with ruminal drinking syndrome. The 5.0-MHz linear transducer was held parallel to the sternum in the left lower thoracic area. The reticular content appears heterogeneous and echoic and the honeycomb-like structure of the mucosa can be seen. 1 Abdominal wall, 2 Spleen, 3 Reticular wall, 4 Reticular content, 5 Honeycomb-like structure of the mucosa, Cr Cranial, Cd Caudal.

The reticulum could be visualised in eight of 10 calves before feeding. The wall of the reticulum appeared as an echoic line, similar to adult cattle [6,8]. The reticular folds were distinct in these eight calves and the honeycomb structure of the mucosa was seen in three (Figure 1). The reticular content appeared as echoic heterogeneous fluid. All contractions were biphasic and similar to those described in mature cattle [6,8]. During the second contraction, the reticulum moved beyond the penetration depth of the transducer. There were 9.4 ± 3.46 (6.0 to 17.0) contractions during a nine-minute period or 1.0 ± 0.38 (0.7 to 1.9) contractions per minute. The first contraction lasted 3.0 ± 0.76 (1.8 to 6.9) seconds and the second 4.9 ± 1.44 (2.0 to 9.0) seconds. The amplitude of the first contraction was 3.6 ± 1.07 (1.5 to 5.1) cm. After incomplete relaxation, the reticulum was 1.5 ± 0.96 (0.0 to 4.0) cm from its original position. The amplitude of the second contraction could not be measured in any of the calves because it exceeded the penetration depth of the transducer. The mean velocity of the first contraction was 2.4 ± 0.63 (1.3 to 3.1) cm/second. The interval between two biphasic contractions was 45.9 ± 22.4 (7.0 to 106.0) seconds. The reticulum could not be seen during ingestion of milk, and was observed in only one calf 15 minutes later. In the latter, the ultrasonographic appearance of the reticulum was the same as before feeding and the variables of reticular motility were within pre-feeding ranges.

### Ultrasonography of the rumen

**Figure 2:**
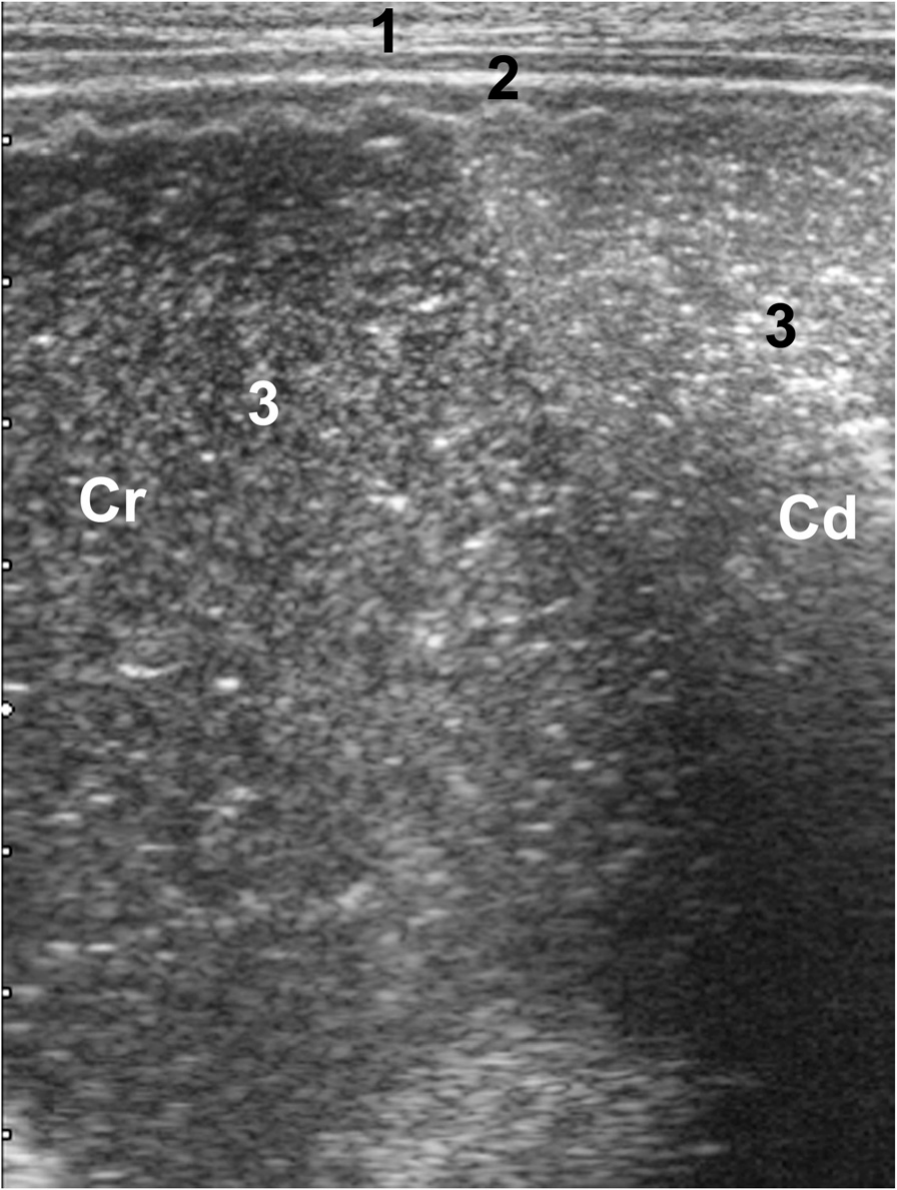
**Ultrasonogram of the ventral sac of the rumen.** Ultrasonogram of the ventral sac of the rumen of a 27-day-old Swiss Braunvieh calf with ruminal drinking syndrome. The 5.0-MHz linear transducer was held parallel to the ventral midline in the left lower abdominal area. The ruminal content appears heterogeneous and echoic. The undulating line medial to the ruminal wall indicates hyperkeratosis. 1 Ventral abdominal wall, 2 Ruminal wall, 3 Ruminal content, Cr Cranial, Cd Caudal.

Before feeding, the rumen appeared as an echoic line with a wall thickness of 2.1 ± 0.61 mm dorsally, 3.5 ± 1.20 mm at the level of the longitudinal groove and 3.2 ± 0.85 mm ventrally. The cranial dorsal blind sac could be seen in six calves and contractions appeared similar to those of adult cattle immediately after the second biphasic reticular contraction [6]. In five of the six calves, the ruminal content could be seen as hypoechoic heterogeneous fluid. In nine calves, a gas cap was seen in the dorsal sac of the rumen, which caused reverberation artifacts running parallel to the ruminal wall, and in one calf, the dorsal sac contained liquid content. In all calves, the ventral sac of the rumen contained echoic heterogeneous fluid (Figure 2).

On the left side, the rumen could be seen from the 7th to the 12th intercostal spaces and in the entire flank region (Table 1); it was seen in all calves in the 9th to 12th intercostal spaces. The dorsal visible limit of the rumen ran parallel to the lungs from cranioventral to caudodorsal. Because of superimposition of the lungs, the dorsal visible limit of the rumen was furthest from the midline of the back (30.0 ± 8.89 cm) in the 7th intercostal space. This distance became progressively smaller caudally because less of the rumen was obscured by the lungs. The minimum distance between the rumen and the midline of the back occurred in the cranial flank and was 12.0 ± 1.66 cm. The distance between the ventral visible limit of the rumen and the midline of the back was similar in all examined locations and varied from 36.3 ± 10.41 cm in the 7th intercostal space to 32.2 ± 9.34 cm in the 10th intercostal space. The visible size of the rumen was largest in the caudal flank at 21.9 ± 10.17 cm. There was a marked decrease in size cranially because of superimposition of the lungs; it was only 6.3 ± 4.73 cm in the 7th intercostal space. The longitudinal groove was visible in all calves and its distance from the midline of the back varied from 25.0 ± 4.54 to 30.0 ± 4.32 cm. The dorsal sac of the rumen was largest at 15.5 ± 6.40 cm in the caudal flank and smallest at 5.8 ± 2.87 cm in the 8th intercostal space, and could not be seen in the 7th intercostal space because of the lungs. The size of the ventral sac was similar in all examined locations and varied from 8.3 ± 4.81 to 10.2 ± 6.83 cm. In five calves, there were visible changes during and after feeding. The milk entering the rumen could be seen as hyperechoic fluid mixing with the fluid already present. On the right side, the rumen was seen in only two calves in the 11th and 12th intercostal spaces and cranial flank.

**Table 1:** Visibility, localisation and size of the rumen in 10 calves with ruminal drinking syndrome (mean, sd, min., max.)

**Variable**	**Intercostal spaces**						**Flank**	
	**7**	**8**	**9**	**10**	**11**	**12**	**Cranial**	**Caudal**
Number of calves	3	8	10	10	10	10	9	7
Dorsal visible limit of rumen (cm)	30.0 ± 8.89	24.5 ± 3.34	19.5 ± 3.21	16.2 ± 2.62	13.9 ± 1.79	12.1 ± 1.66	12.0 ± 1.66	12.1 ± 2.73
	(23.0 – 40.0)	(20.0 – 28.0)	(15.0 – 26.0)	(12.0 – 22.0)	(11.0 – 17.0)	(9.0 – 15.0)	(8.0 – 13.0)	(6.0 – 14.0)
Ventral visible limit of rumen (cm)	36.3 ± 10.41	36.1 ± 7.61	32.2 ± 9.34	33.9 ± 9.18	34.7 ± 9.73	33.3 ± 10.80	33.1 ± 12.45	34.0 ± 11.46
	(28.0 – 48.0)	(29.0 – 49.0)	(20.0 – 48.0)	(21.0 – 48.0)	(22.0 – 51.0)	(17.0 – 51.0)	(17.0 – 53.0)	(18.0 – 49.0)
Size of rumen (cm)	6.3 ± 4.73	11.6 ± 8.53	12.8 ± 9.74	17.7 ± 9.60	20.8 ± 9.24	21.2 ± 10.25	21.1 ± 11.78	21.9 ± 10.17
	(1.0–10.0)	(3.0 – 25.0)	(3.0 – 30.0)	(6.0 – 36.0)	(11.0 – 36.0)	(8.0 – 39.0)	(8.0 – 41.0)	(12.0 – 36.0)
Distance of longitudinal groove from midline of back (cm)	-	30.0 ± 4.32	25.0 ± 4.54	25.6 ± 4.69	25.6 ± 5.02	25.7 ± 4.68	27.8 ± 4.55	28.8 ± 6.08
		(26.0 – 36.0	(18.0 – 33.0)	(21.0 – 35.0)	(20.0 – 34.0)	(21.0 – 32.0)	(23.0 – 33.0)	(23.0 – 34.0)
Size of dorsal sac of rumen (cm)	-	5.8 ± 2.87	6.8 ± 3.85	9.6 ± 5.10	12.4 ± 5.50	12.7 ± 5.79	15.0 ± 4.80	15.5 ± 6.40
		(2.0 – 8.0)	(2.0 – 12.0)	(3.0 – 17.0)	(7.0 – 24.0)	(7.0 – 21.0)	(10.0 – 20.0)	(9.0 – 21.0)
Size of ventral sac of rumen (cm)	-	10.0 ± 6.22	9.0 ± 5.70	9.8 ± 4.89	8.3 ± 4.81	9.7 ± 5.71	10.2 ± 6.83	9.0 ± 4.32
		(3.0 – 17.0)	(3.0 – 18.0)	(4.0 – 20.0)	(2.0 – 17.0)	(4.0 –20.0)	(4.0 – 21.0)	(5.0 – 15.0)

### Ultrasonography of the omasum

**Figure 3:**
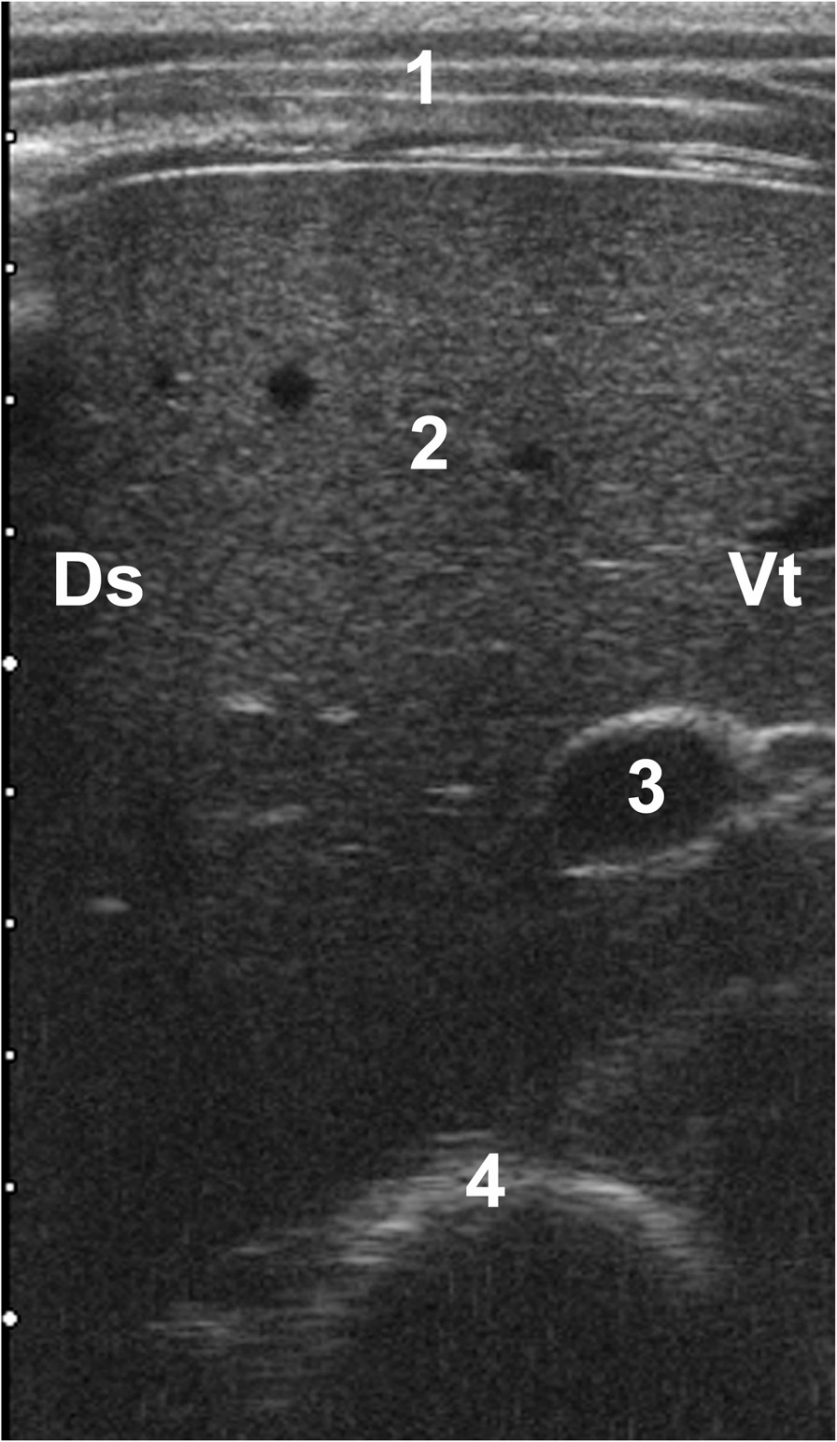
**Ultrasonogram of the omasum.** Ultrasonogram of the omasum and liver of a 27-day-old Swiss Braunvieh calf with ruminal drinking syndrome. The 5.0-MHz linear transducer was held on the right side parallel to the ribs in the 8th intercostal space. The omasum is seen medial to the liver as a crescent-shaped echo with the convex side toward the transducer. 1 Abdominal wall, 2 Liver, 3 Portal vein, 4 Omasum, Ds Dorsal, Vt Ventral.

The omasum could be seen in seven of ten calves on the right side as a crescent-shaped echoic line 2.7 ± 0.85 mm thick medial to the liver (Figure 3). Only the wall closest to the transducer was visible. The bases of the omasal leaves were seen in two calves. The omasal content and the omasal wall furthest from the transducer could be seen in one calf, which had a small amount of echoic liquid content. Omasal motility was not seen during the four-minute observation period in any of the calves. The omasum was seen in one intercostal space in one calf, in two intercostal spaces in five calves and in three adjacent intercostal spaces in one calf. The ultrasonographic appearance of the omasum did not change during and after feeding. The dorsal visible limit of the omasum ran from cranioventral to caudodorsal and was furthest from the midline of the back (40.0 cm) in the 6th intercostal space (Table 2). This distance became progressively smaller caudally and measured 24.5 ± 3.54 cm in the 9th intercostal space. The ventral visible limit of the omasum had a similar course; its distance from the midline of the back was 44.5 cm in the 6th and 29.5 ± 6.36 cm in the 9th intercostal space. The size of the omasum was largest at 5.2 ± 2.40 cm in the 8th intercostal space and became progressively smaller cranially and caudally. The size was smallest at 4.4 ± 0.55 cm in the 7th intercostal space.

**Table 2:** Visibility, localisation and size of the omasum in 10 calves with ruminal drinking syndrome (mean, sd, min., max.)

**Variable**	**Intercostal spaces**			
	**6**	**7**	**8**	**9**
Number of calves	1	5	6	2
Dorsal visible limit of omasum (cm)	40.0	30.4 ± 5.27	25.3 ± 3.33	24.5 ± 3.54
		(25.0 – 39.0)	(20.0 – 30.0)	(22.0 – 27.0)
Ventral visible limit of omasum (cm)	44.5	34.8 ± 5.59	30.5 ± 5.01	29.5 ± 6.36
		(29.0 – 44.0)	(24.0 – 38.0)	(25.0 – 34.0)
Size of omasum (cm)	4.5	4.4 ± 0.55	5.2 ± 2.40	5.0 ± 2.83
		(4.0 – 5.0)	(2.0 – 8.0)	(3.0 – 7.0)

### Ultrasonography of the abomasum

**Figure 4:**
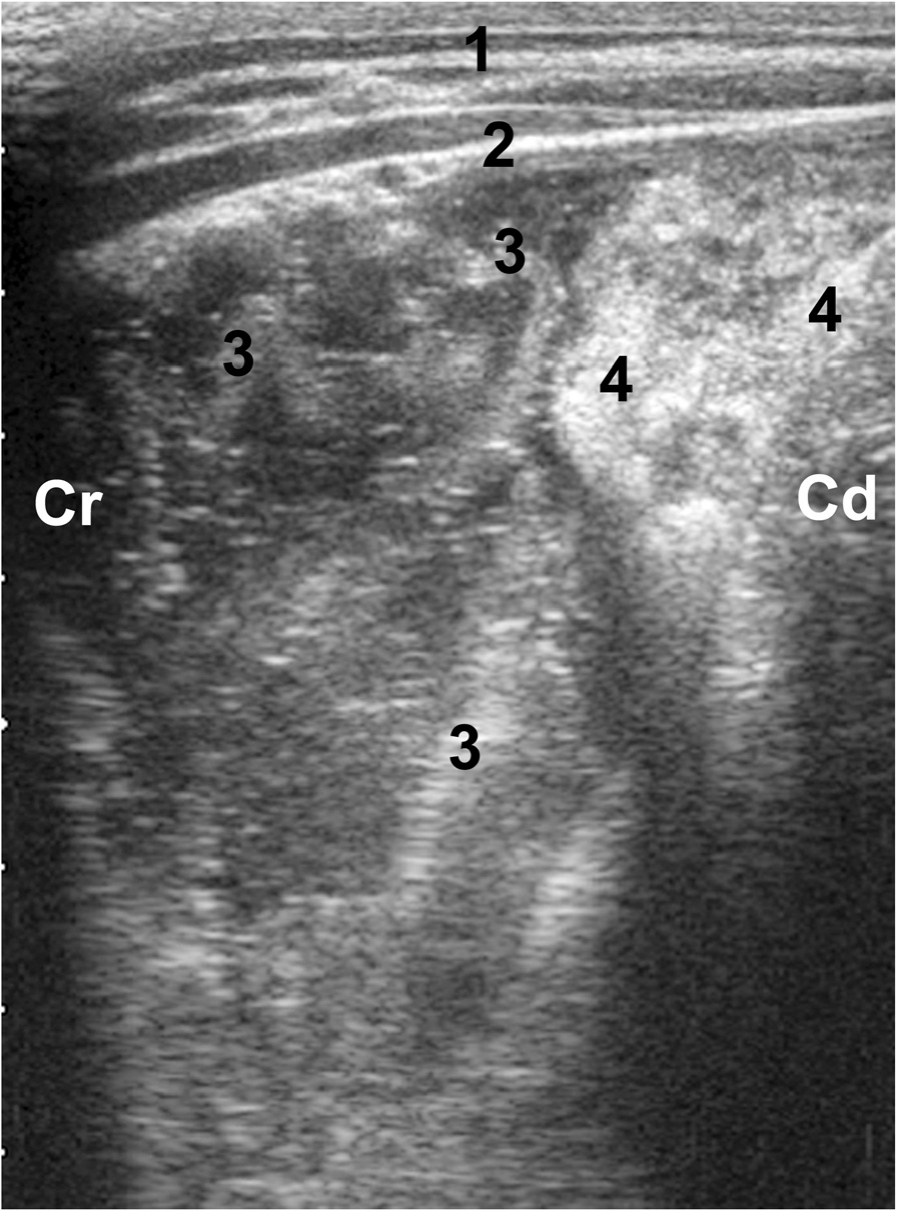
**Ultrasonogram of the abomasum.** Ultrasonogram of the abomasum of a 17-day-old calf with ruminal drinking syndrome. The 5.0-MHz linear transducer was held parallel to the ventral midline in the left lower abdominal area. The abomasal content appears heterogeneous and echoic, and abomasal leaves are seen as curved structures. 1 Ventral abdominal wall, 2 Abomasal wall, 3 Abomasal leaves, 4 Ingesta, Cr Cranial, Cd Caudal.

The abomasum was visible on the left and right of the ventral midline immediately caudal to the xyphoid before feeding in nine calves (Figure 4). It lay adjacent to the abdominal wall along the ventral midline in all calves and was 22.2 ± 8.29 cm (15.0 to 41.0 cm) long. The wall appeared as an echoic line 1.7 ± 0.68 mm thick. The abomasal folds were distinct in eight calves. The ingesta were heterogeneous in all calves (Table 3) and were largely liquid in six calves and contained equal liquid and firm parts in the remaining four. The liquid was hypoechoic in nine calves and echoic in one. There were solid particles in the fluid phase, which were smaller than 1 cm in two calves and between 1 cm and 5 cm in eight. The pylorus could be seen in six calves on the right side. The lateral expansion of the abomasum from the median to the left side varied from 8.7 ± 3.03 cm in the 6th intercostal space to 13.8 ± 6.85 cm in the 12th intercostal space (Table 4). Lateral expansion to the right was smallest at 4.3 ± 6.01 cm in the caudal flank and largest at 11.3 ± 3.81 cm in the 11th intercostal space. The extent of lateral expansion to the left and right did not differ.

**Table 3:** Abomasal content of 10 calves with ruminal drinking syndrome before, during and 15 minutes after ingestion of milk

**Variable**		**Time relative to feeding**		
		**Before**	**During**	**15 minutes after**
Abomasal leaves distinct		8	9	7
Content homogeneous		0	9	0
Content heterogeneous		10	1	10
Milk recognisable		0	10	10
Milk clotting recognisable		0	0	10
Liquid hypoechoic		9	5	9
Relationship between solid and liquid contents	mostly liquid	6	9	4
	liquid and solid	4	1	3
	mostly solid	-	-	3
Size of solid particles	≤ 1.0 cm	2	5	1
	1.01 – 5.0 cm	8	5	5
	> 5.0 cm	-	-	4

**Table 4:** Expansion of the abomasum from the ventral midline to the left and right in ten calves with ruminal drinking syndrome before ingestion of milk (mean, sd, min., max.)

**Localisation**	**Expansion to the left (cm)**		**Expansion to the right (cm)**	
	**Number of calves**	**Mean ± sd (range)**	**Number of calves**	**Mean ± sd (range)**
6. ICS	7	8.7 ± 3.03	6	6.5 ± 6.24
		(4.5 – 13.0)		(0.0 – 14.5)
7. ICS	10	11.5 ± 5.13	9	8.4 ± 4.84
		(3.0 – 19.5)		(0.0 – 15.5)
8. ICS	10	12.8 ± 5.63	9	9.3 ± 4.34
		(4.0 – 22.0)		(0.0 – 14.0)
9. ICS	10	11.8 ± 5.09	9	10.0 ± 5.08
		(4.0 – 18.0)		(0.0 – 17.5)
10. ICS	10	11.9 ± 6.09	9	9.83 ± 4.62
		(3.5 – 19.0)		(0.0 – 14.0)
11. ICS	9	10.8 ± 7.30	8	11.3 ± 3.81
		(0.0 – 20.0)		(4.5 – 15.5)
12. ICS	8	13.8 ± 6.85	7	10.4 ± 4.74
		(4.5 – 24.0)		(0.0 – 14.0)
Cranial flank	5	13.6 ± 8.51	4	10.3 ± 3.80
		(4.5 – 23.5)		(5.0 – 13.5)
Caudal flank	4	13.5 ± 4.81	2	4.3 ± 6.01
		(8.5 – 20.0)		(0.0 – 8.5)

*ICS* Intercostal space.

The milk entering the abomasum was visible in all calves. Immediately after feeding, the abomasal content was homogeneous and echoic and the abomasal folds were distinct in nine calves. There were no signs of milk clotting (consolidation of the echoic material) at the end of feeding in any of the calves. There were distinct changes in the abomasal content 15 minutes after feeding; in all calves the content was heterogeneous but the abomasal folds were only visible in seven calves. Milk clots were seen in all calves as hyperechoic clumps as described previously [15,17]. The clumps were less than 1 cm, between 1 and 5 cm and larger than 5 cm in one, five and four calves, respectively. The abomasal content was mainly liquid in four calves, had similar proportions of liquid and solid components in three and was mainly solid in three others.

## Discussion

The same ultrasonographic technique used for examination of the reticulum in adult cattle [6] can be used in young calves. In contrast to cows but similar to healthy milk-fed calves [19,20], the reticulum could be visualised only in 8 of the 10 calves. This is likely due to its relatively small size in calves and because it is not situated on the ventral abdominal wall. During feeding and 15 minutes later, the reticulum could not be seen in nine of ten calves because it was displaced craniodorsally by the expanding abomasum and superimposed by the lungs. At these stages of feeding, the reticulum could not be seen in any of ten healthy milk-fed calves [19]. The reticular wall of ruminal drinkers appeared as a smooth echoic line similar to healthy milk-fed calves and adult cattle, but the ultrasonographic appearance of the reticular content differed. Unlike healthy calves, in which the content could not be imaged [19,20], the reticulum of the eight ruminal drinkers in which the organ could be visualised had a heterogeneous echoic liquid content. Furthermore, reticular folds could be seen in eight ruminal drinkers; these structures were only seen vaguely in one healthy calf [19,20]. The honeycomb-like structure of the reticular mucosa was visible in three ruminal drinkers, but not in healthy calves [19,20]. Thus, the analysis of the reticular content is useful for differentiating ruminal drinkers and healthy milk-fed calves. Reticular motility in ruminal drinkers with largely acidic and liquid content was comparable to that of healthy milk-fed calves [19,20], which was surprising in view of reports that acidic rumen juice inhibits forestomach motility [2,4].

The rumen was easily visualised in all calves although in relative terms it is much smaller in calves than in mature cattle. Milk in the rumen was readily detected ultrasonographically; it was at the level of the longitudinal groove in three calves and even filled the dorsal sac of the rumen in one. In healthy calves, a gas cap was seen in the dorsal sac of the rumen, which caused a reverberation artifact running parallel to the ruminal wall [19,20]. The transition from gas to ingesta was characterized by an abrupt end of the reverberation artifact lines. Differentiation of the ingesta and the ventral fluid phase was not possible in any of the calves because of gas. The fluid phase could not be seen in any of the healthy calves examined [19,20]. From CT-examinations it is known, that the fluid phase of healthy calves has a size of only few centimetres [22]. Interestingly the ruminal wall was thicker in ruminal drinkers than in healthy milk-fed calves near the longitudinal groove (3.5 versus 2.2 mm) and in the ventral ruminal sac (3.2 versus 1.7 mm) [19,20] but not in the dorsal ruminal sac. Our interpretation of this was that hyperkeratotic parakeratosis and ruminitis, induced by butyric and lactic acid [1], accounted for the differences, rather than a difference in age of the calves.

The omasum of ruminal drinkers and healthy milk-fed calves did not differ ultrasonographically [19]. As in cows [10] the omasal content could not be visualised in ruminal drinkers. In another study involving calves 12 hours to 2 weeks of age, the omasal leaves appeared as distinct echoic structures within anechoic omasal content [14].

The size of the abomasum in ruminal drinkers was the same as in healthy milk-fed calves [19]. In adult cattle the abomasal content is diffusely echoic with hyperechoic stippling, and remains unchanged during and after feeding [11]. Similar to healthy milk-fed calves [14,15,17] the ultrasonographic appearance of the abomasal content of ruminal drinkers varied considerably with the ingestion of milk.

## Conclusions

This study has shown that ultrasonography is useful for detecting milk in the reticulum and rumen of calves with ruminal drinking syndrome. The ultrasonographic appearance of the omasum and abomasum did not differ between ruminal drinkers and healthy milk-fed calves.

## Competing interests

The authors declare that they have no competing interests.

## Authors’ contributions

UB initiated and planned the study and he prepared the manuscript. AG carried out the ultrasonographic examinations under supervision of UB. Both authors have read and approved the manuscript.

## Source of fundings

This study was financed by the University of Zurich, Switzerland.
